# Editorial: Biotechnology frontiers in pharmacology

**DOI:** 10.3389/fphar.2026.1824432

**Published:** 2026-04-14

**Authors:** Mónica Vieira, Cledir Santos, Ricardo Ferraz

**Affiliations:** 1 RISE-Health, Centro de Investigação em Saúde Translacional e Biotecnologia Médica (TBIO), Escola Superior de Saúde, Instituto Politécnico do Porto, Porto, Portugal; 2 Scientific and Technological Bioresources Nucleus (BIORENUFRO), Universidad de La Frontera, Temuco, Chile; 3 Postgraduate Program in Biotechnology, Federal University of Technology – Paraná, Ponta Grossa, Brazil; 4 LAQV-REQUIMTE, Departamento de Química e Bioquímica, Faculdade de Ciências da Universidade do Porto, Porto, Portugal

**Keywords:** bioactive compounds, biomarkers, medicinal biotechnology, one health, pharmacology

Biotechnology has become a crucial player in today’s biomedical research. By studying the capabilities of living systems, including microorganisms, plants, and animal-derived cellular platforms, biotechnological approaches enable the production of a wide array of biologically active substances and diagnostic tools. These range from therapeutic molecules and biological markers to complex therapeutic platforms with applications across medicine, agriculture, and environmental health. In recent years, the close interaction between biotechnology and pharmacology has gained relevance, as advances in molecular biology, bioengineering, and pharmaceutical technology continue to reshape how therapeutic agents are discovered, optimized, and delivered.

In pharmacological research, biotechnology now contributes much more than the initial identification of bioactive molecules. It informs the design of advanced delivery systems, supports the characterization of drug–target interactions, and helps the development of precise therapeutic strategies. In parallel, the growing acknowledgement that human, animal, and environmental health are connected has reinforced the importance of adopting a broader translational perspective. From this viewpoint, biotechnology provides a versatile framework through which pharmacological innovation can address complex and multifactorial health challenges.

The Research Topic *Biotechnology Frontiers in Pharmacology* gathered emerging work in the frontiers of Medicinal Biotechnology and Pharmacology. The goals of this collection were not only to present recent advances in this area but also to show how diverse biotechnological strategies can converge toward common pharmacological objectives, including the identification of novel therapeutic agents, the optimization of drug delivery systems, and the translation of experimental findings into clinically meaningful applications.

In this Research Topic, the articles presented in this collection concern the development and clinical translation of advanced therapeutic platforms. A systematic review addressing the global landscape of clinical research in cell therapy provides a comprehensive overview of ongoing clinical trials, marketed products, and evolving regulatory frameworks (Wang et al.). The authors highlighted the important role of both the USA and China in oncology therapies, particularly in the use CAR-T cells, while Europe is working in hematopoietic stem cell applications. This development is very dependent on regulatory frameworks, such as those of the FDA and EMA, for the approval and accessibility of these advanced treatments. By examining current clinical pipelines and regulatory trends, this analysis offers valuable insight into how complex biological therapies are advancing toward broader clinical implementation (Wang et al.).

Progress in pharmaceutical biotechnology with the optimization of drug delivery technologies is also emphasized in the present collection. The work “*Anti-convulsant efficacy of long-acting injectable cannabidiol formulation (IVL5005) in the pentylenetetrazol-induced convulsions, with pharmacokinetic characterization*” demonstrates how formulation strategies can influence pharmacological properties of the drug. Through pharmacokinetic characterization and assessment in a pentylenetetrazol-induced seizure model, the study demonstrates how sustained-release delivery systems may improve therapeutic exposure and potentially enhance treatment efficacy. Such work highlights the importance of integrating formulation science, pharmacokinetics, and pharmacodynamics in the design of next-generation therapeutics (Youm et al.). The other research article “*In Vitro antiproliferative potential of essential oil extract from Carica papaya L. seeds against cervical cancer*” shows the importance of the evaluation of biologically derived molecules with anticancer activity. Such kind of research emphasizes the importance of combining biological screening with chemical characterization and mechanistic investigation to support the translational potential of candidate compounds (Nady et al.).

Another important characteristic of biotechnology-driven pharmacology concerns the identification and characterization of bioactive molecules with therapeutic potential. Natural products remain an undeniable source of relevant compounds, particularly when their chemical structures and mechanisms of action can clearly be defined. The review focusing on *Betulinic acid and obesity-related disorders* provides a detailed overview of the pharmacological properties of this triterpenoid compound and its possible implications on metabolic disease (Azevedo et al.). The work reviews the most continued relevance of chemically characterized natural compounds in drug discovery (Azevedo et al.).

Taken together, the contributions gathered in this Research Topic reflect the broad spectrum of strategies shaping biotechnology-driven pharmacology, nowadays. The contributions cover emerging therapeutic modalities such as cell-based therapies, advances in drug formulation and delivery, as well as the continued exploration of bioactive molecules derived from natural sources. Despite their diversity, these studies share a common objective: to improve the understanding of pharmacological mechanisms while advancing the development of more effective therapeutics.

The future in this area will likely depend on the integration of several rapidly evolving technologies. Omics-based methodologies, advanced analytical platforms, and artificial intelligence–assisted drug discovery are already changing how pharmacologically active molecules are identified and optimized. At the same time, the discovery and validation of reliable biomarkers will continue to be necessary for improving disease diagnosis, monitoring therapeutic responses, and guiding precision medicine approaches. In this sense, the ongoing evolution of regulatory frameworks capable of accommodating increasingly complex biotechnological products is also crucial.

In conclusion, the works presented in this Research Topic demonstrate how biotechnology continues to expand the boundaries of pharmacology. By bridging molecular discovery, technological innovation, and translational research, biotechnology is playing an increasingly central role in shaping the future of therapeutic development and in addressing pressing global health challenges.

